# Vitamin C: a misunderstood ally?

**DOI:** 10.1186/s13054-020-2725-x

**Published:** 2020-01-07

**Authors:** Jiajia Ren, Xuting Jin, Ya Gao, Ruohan Li, Jiamei Li, Jingjing Zhang, Xiaochuang Wang, Gang Wang

**Affiliations:** Department of Critical Care Medicine, The Second Affiliated Hospital of Xi’an Jiaotong University, Xi’an, 710004 Shaanxi Province China

As an important antioxidant, vitamin C deficiency has frequently been observed in critically ill patients [1]. The treatment with early high-dose intravenous vitamin C has shown beneficial effects on patients with sepsis, septic shock, and myocardial ischemia in both preclinical and clinical studies [2]. Recently, 2 original articles caught my attention. In December 2019, a nationwide cohort study involving 2713 patients reported in *Critical Care* demonstrated that high-dose vitamin C therapy was associated with reduced mortality in patients with severe burns [3]. The pleiotropic effects of vitamin C may result from its protection against oxidative stress-mediated cell damage and organ dysfunction [1]. However, the CITRIS-ALI trial, reported earlier by Fowler and colleagues in *JAMA*, October 2019, showed an inconsistent result. In this study, 167 adults with sepsis and acute respiratory distress syndrome were randomized to receive either high-dose vitamin C or placebo for 96 h, and the primary outcomes including modified Sequential Organ Failure Assessment (mSOFA) scores were not statistically different between the groups [4], despite the expected protective effects of vitamin C against multiple organ failure, inflammation, and endothelial injury as shown in their phase I safety trial [5].

After scrutinizing the results in the CITRIS-ALI trial, we proposed that a survivorship bias may have contributed to the conflicting result. As we noted from their results (Fig. 3, page 1268), there were sudden increases in the overall mortality probability from day 0 to day 7 in the placebo group. Thus, we performed further analyses according to Fig. 3 and discovered that the overall mortalities in the placebo group had more intensive increases on day 2 (10.8 vs. 1.2%; *P* = 0.021), day 3 (14.5 vs. 2.4%; *P* = 0.005), and day 4 (19.3 vs. 3.6%; *P* = 0.001) than those in the vitamin C group (shown in Table 1). Those sudden increases in mortality in the placebo group may result from the deteriorated conditions of certain patients, leaving the survived patients with less severe conditions for statistical analyses at the primary end points. Therefore, when the mSOFA at 96 h and the levels of C-reactive protein and thrombomodulin at 168 h were evaluated, the survivorship bias rendered the differences between the vitamin C group and placebo group uncomparable, causing decreased reliability of the results. Nevertheless, a positive effect of vitamin C still gleams behind the results presented, as a misunderstood ally.

**Table 1:** A rough estimate of mortalities from day 0 to day 7

Day		1	2	3	4	5	6	7
Placebo (*n* = 83)	No. at risk	82	74	71	67	64	61	59
	Mortality (%)	1 (1.2)	9 (10.8)	12 (14.5)	16 (19.3)	19 (22.9)	22 (26.5)	24 (28.9)
Vitamin C (*n* = 84)	No. at risk	84	83	82	81	80	78	74
	Mortality (%)	0 (0)	1 (1.2)	2 (2.4)	3 (3.6)	4 (4.8)	6 (7.1)	10 (11.9)

The overall mortalities in the placebo group had more intensive increases on day 2 (10.8 vs. 1.2%; *P* = 0.021), day 3 (14.5 vs. 2.4%; *P* = 0.005), and day 4 (19.3 vs. 3.6%; *P* = 0.001) compared with those in the vitamin C group

## Data Availability

Not applicable.

## Abbreviations

mSOFA scores: Modified Sequential Organ Failure Assessment scores
